# Restrictions to Pharmacy Ownership and Vertical Integration in Estonia—Perception of Different Stakeholders

**DOI:** 10.3390/pharmacy4020018

**Published:** 2016-04-19

**Authors:** Marit Gross, Daisy Volmer

**Affiliations:** Institute of Pharmacy, Faculty of Medicine, University of Tartu, 1 Nooruse Str, Tartu 50411, Estonia; marit.gross@ut.ee

**Keywords:** pharmaceutical policy, restrictions, ownership of community pharmacies, vertical integration, Estonia

## Abstract

Objectives: From 2020, the ownership of community pharmacies in Estonia will be limited to the pharmacy profession, and the vertical integration of wholesale companies and community pharmacies will not be allowed. The aim of this study was to evaluate the perception of different stakeholders in primary healthcare toward the new regulations of the community pharmacy sector in Estonia. Methods: A qualitative electronic survey was distributed to the main stakeholders in primary healthcare and higher education institutions providing pharmacy education (*n* = 40) in May 2015. For data analysis, the systematic text condensation method was used. Results: The study participants described two opposing positions regarding the development of community pharmacies in the future. Reform supporters emphasized increased professional independence and more healthcare-oriented operation of community pharmacies. Reform opponents argued against these ideas as community pharmacists do not have sufficient practical experience and finances to ensure sustainable development of the community pharmacy sector in Estonia. Conclusion: Based on the current perception of all respondents, the future operation of the community pharmacy sector in Estonia is unclear and there is urgent need for implementation criteria for the new regulations.

## 1. Introduction

Community pharmacies have an important function within the healthcare system, providing the dispensing of and counseling about medicinal products, as well as self-medication and health promotion. Due to the relevant role that pharmacists play in the delivery of healthcare, community pharmacies in the majority of cases are highly regulated in most European countries [1]. The following restrictions have been established individually or in combination:

- ownership—limited to the pharmacy profession, limited number of pharmacies (horizontal integration), limited to prescribers, manufacturers, and wholesalers (vertical integration);
- demographic and geographic restrictions for opening a new pharmacy;
- pharmacy monopoly for sale of prescription and (non-prescription) medicines;
- standard requirements for marketing authorization of medicines;
- transparent pricing system (e.g., fixed markups and reimbursement principles) of medicines [1,2].

There are several examples of countries in Europe (e.g., Germany, Spain, Hungary, Austria, and Finland) where the ownership of community pharmacies is limited to the pharmacy profession [3,4]. On the other hand, the UK, Ireland, the Netherlands, Norway and Iceland serve as examples of a liberalized community pharmacy sector, having mostly no restricting criteria for ownership, establishment or location of community pharmacies [1,2]. The advocates of both systems promise to guarantee the provision of quality community pharmacy services for customers. In countries with strictly regulated community pharmacies, the independence of the service provider and financial support from governments is emphasized to counter the inefficiencies of the monopoly system. In countries with a liberal community pharmacy system, they rely on increased competition, the lowering of healthcare expenditures, and better access to community pharmacy services due to the opening of new outlets [2]. Lluch and Kanavos demonstrated that there are useful lessons to learn from both systems: liberal countries could look into the policies applied in strictly regulated countries that increase the equity of community pharmacies, whereas regulated countries could adopt some of the policies from liberal countries to increase efficiency in the system [5].

Estonia has been a country with a liberal pharmaceutical policy for more than 20 years (Table 1). The pharmaceutical sector in Estonia was permeated by substantial reforms in the early 1990s. It was necessary to establish pharmaceutical regulatory authorities, create a legislative framework, develop a reimbursement system for medicines, and rearrange the community pharmacy sector [6]. Currently, the regulatory framework for the pharmaceutical sector is based on the Medicinal Products Act (first adopted in 1996 and revised in 2005) [7], and the Health Insurance Act [8].

Privatization of the community pharmacy sector in Estonia began immediately after the regaining of independence in 1991. The opening, operation and management of community pharmacies are strictly regulated by the Medicinal Products Act. Since 1996, however, the ownership of community pharmacies has no longer been limited to the pharmacy profession. The reasoning behind liberalization was mostly connected to economical needs and less connected to improved patient care.

Vertical and horizontal integration of community pharmacies started to emerge in the second half of the 1990s. The liberal system has led to a rapid growth in the number of community pharmacies, from about 250 in 1993 to 476 (310 main pharmacies with 166 structural units) in 2015. Currently, approximately 90% of community pharmacies (the majority operating in larger towns) are joined through ownership or partner status to chains what are mostly connected to wholesale companies. This is one of the reasons why the vast majority of pharmacies buy most medicinal products from certain wholesalers. In the current situation, competition in the pharmaceutical wholesale and retail market is limited and new competitors find it difficult to enter the market [6,10,11].

Demographic and geographic restrictions to the opening of new entities existed between 2006–2013, but did not fulfill their purpose about a more even distribution of pharmacies in rural areas. Even contrary, since 2006, the number of community pharmacies has decreased by 5% in towns, and by 12% in the countryside. In December 2013, the State Court repealed the establishment criteria for community pharmacies [10]. Temporary restrictions were composed in 2014, and in March 2015, the ownership restriction came into force. After the five-year transition period in 2020, only pharmacists can be owners (hold more than 50% of pharmacy shares) of up to four community pharmacies, and work as pharmacy managers in one of the owned pharmacies. Manufacturers, wholesale companies and prescribers are not allowed to be shareholders in community pharmacies. The last described restriction came into force in 2014 with a transition period of five years [7]. In 2014, pharmacists legally became healthcare professionals [12]. However, community pharmacy services have not yet been classified as a healthcare service in Estonia today.

## 2. Objectives

The aim of this qualitative electronic survey was to evaluate the perceptions towards the new regulations of the community pharmacy sector among different stakeholders in and connected to the primary healthcare of Estonia.

## 3. Methods

### 3.1. Study Design and Sample

**Figure 1:**
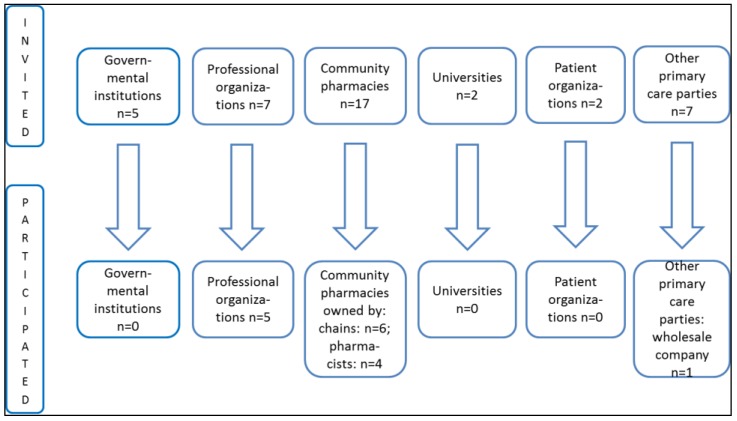
Participation of different stakeholders in the survey.

A qualitative electronic survey using the web platform Google Sheets was used for data collection. The survey was conducted in May 2015 and forwarded to 40 different parties of primary healthcare in Estonia: governmental institutions, professional and patient organizations, representatives of community (owners-pharmacists and owners-chains) pharmacies and wholesale companies of medicinal products; and universities providing pharmacy education at the Bachelor and Master’s level (Figure 1). The survey was anonymous and participants presenting the position of the represented institution or organization were asked to complete one survey instrument. Two reminders were forwarded to the respondents during the survey with the request to complete the survey instrument.

Present research conforms to the legal and ethical standards of Estonia. Separate approval from the ethics committee was not required for this type of study.

### 3.2. Survey Instrument

Publicly available information about (political) discussions and positions of different stakeholders before and after changes in pharmaceutical legislation was used for the development of the survey instrument. The survey was planned and the questions were self-designed by the panel of representatives from the University of Tartu, the Estonian State Agency of Medicines, and the Estonian Pharmacy Association.

The survey instrument consisted of the following open-ended questions and respondents were asked to justify their responses:

(1) How could the transition of pharmacy ownership be organized to satisfy all parties involved? Should the government provide financial support to pharmacists who want to open or buy a pharmacy?
(2) What impact could the prohibition of vertical integration have on the pharmacy sector?
(3) Will the new regulation increase or decrease competition in the community pharmacy sector? Will new companies enter the pharmaceutical wholesale market after the prohibition of vertical integration?
(4) Will the new regulation increase or decrease the number of community pharmacies? How will the situation change for community pharmacies in rural areas?
(5) Will the new regulation change the quality of community pharmacy services? Should community pharmacy services be classified as healthcare services?
(6) Will the pricing of medicines and wages for community pharmacy professional staff change with the new regulation?
(7) Will the professional roles and responsibilities of a pharmacist and an assistant pharmacist change with the new regulation?
(8) Should pharmacy education be updated according to the new regulation?

For content and face validity, the survey instrument was evaluated by a small sample (*n* = 5) of representatives from a governmental institution, a professional organization, a wholesale company, and practicing pharmacists.

### 3.3. Data Analysis

For data analysis, a systematic text condensation method was used. The method is a descriptive and explorative method for the thematic analysis of different types of qualitative data: interview studies, observational studies, and written texts.

The method includes four steps:

(1) total impression—researcher reads the entire description or all results to get a general understanding about the topic;
(2) identifying and sorting meaning units—the researcher identifies and organizes the data by meaning units that are related to the study question;
(3) condensation—the researcher examines meaning units one by one to get a detailed understanding of the content of every unit; the data is decontextualized;
(4) synthesizing—the researcher condenses information received from meaning units into a consistent statement/results, and the data is put back into context [13].

Described structure was used to analyze all questions and each question was analyzed separately. If applicable, meaning units were divided and condensed by positive/negative/neutral perceptions of the respondents.

## 4. Results

This study, using a qualitative electronic survey, was aimed for evaluating the perception of the main stakeholders in primary healthcare as well as the representatives of higher education institutions in Estonia about ownership restrictions of community pharmacies and restrictions of vertical integration that will come into force in 2020. The results are based on 16 completed survey instruments from pharmacy professional organizations (*n* = 5), community pharmacies owned by pharmacists (*n* = 4) and chains (*n* = 6), and from a wholesale company of medicines (*n* = 1) (Figure 1).

### 4.1. Community Pharmacy Service as a Healthcare Service

All respondents agreed on the need to classify community pharmacy services as healthcare services in the future: “Community pharmacy services could reduce or divide the work load of family physicians and nurses, and should therefore clearly be a part of the primary care services in the future.”

Respondents emphasized the strong need to increase collaboration between physicians and pharmacists. Community pharmacies should be re-designed to ensure more private and patient-centered communication, and the government should be involved in the development of extended services at community pharmacy. “There should be a list of traditional and extended community pharmacy services. This document could serve as a basis for future negotiations with the Estonian Health Insurance Fund about the remuneration of extended community pharmacy services.”

### 4.2. The Impact of New Regulations

All respondents agreed that there is an urgent need for information about the transition conditions of community pharmacy ownership from pharmacy chains to pharmacists. They described the increased role of the government in specifying the structure of the community pharmacy sector (e.g., the number and geographical location of community pharmacies), and in making it easier for community pharmacists to receive bank loans. In addition, a longer and more gradual transition period (between 7–10 years) was suggested as it would be impossible to complete the planned reforms in less than five years. 

The impact of the new regulations on the community pharmacy sector in Estonia was mostly described by positive or negative scenarios. All respondents agreed that the number of community pharmacies could decrease in towns and in rural areas—pharmacists do not have the finances and interest to buy and operate non-profitable entities. While the smaller number of pharmacies in towns could lead to the opening of larger pharmacies and encourage the development of extended services, it could cause problems with access to medicines in rural areas. Despite the possible emergence of large pharmacies in towns, the system for the provision and development of community pharmacy services and continuing professional education of community pharmacists remains unclear.

The restriction of vertical integration “will increase professional independence of community pharmacists and decrease commercial influence on operation of community pharmacies.” According to another opinion, “The new regulation would jeopardize the retail sale of medicines, the maintenance and development of the current community pharmacy system needs financial support from the wholesale sector.”

Changes in the pricing of medicines were directly connected with restrictions of vertical integration. “Opening the pharmaceutical market will enable new wholesale companies to enter the pharmaceutical market in Estonia and increased competition would result in the decrease of medicine price,” future owners concluded. Current owners gave a completely opposing description: “The new regulation will end the collaboration between the wholesale and retail sector that currently provides efficient discounts on medicine prices”.

Table 2 outlines the most common perceptions about the impact of new regulations on the operation of the community pharmacy system in Estonia.

## 5. Discussion

There is no common pattern for the pharmaceutical retail and wholesale sector in Europe. Based on the trends of the last decade, community pharmacy chains have become more prevalent and vertical integration between wholesalers and retailers has also been on the rise in EU. Some form of a pharmacy chain is allowed in 19 EU countries, and vertical integration is implemented in 10 EU countries [14]. Although some European countries maintain the monopoly status of pharmacists as the owners of community pharmacies, there have been examples of the deregulation of the community pharmacy sector towards a more liberal system, for example in Sweden in 2009 [15].

New regulations of the community pharmacy sector in Estonia aim to reduce the degree of liberalism in pharmaceutical policy. Over the last 20 years, community pharmacies in Estonia have become modern healthcare institutions offering better access to a large selection of medicines and patient-centered services. On the other hand, privatization and the deregulation of pharmacy ownership have resulted in an increased number of new pharmacies, especially in larger towns. This has created a shortage of pharmacists and assistant pharmacists, which could be seen as a limiting or delaying factor in the introduction of novel, extended and patient-centered services at community pharmacies. Decreasing profit margins may threaten the viability of small community pharmacies in rural areas, potentially limiting consumer access to community pharmacy services in the future [16].

In this study, the future operation of a newly regulated community pharmacy sector was unclear for all participants. Reform proponents based their descriptions of future developments mostly on theoretical considerations about management of community pharmacies and emphasized professional ethics and the independence of pharmacists. Without support from the government, however, these ideas would be too declarative and lack actual solutions for practical implementation. Opponents of the reforms combined economical and professional thinking linked to existing practical experience on operation of community pharmacies in their descriptions. However, the ideas described were mostly based on the defense and approval of the current situation and not really open to possible new developments or collaboration with new owners.

New regulations of the community pharmacy sector have brought to light several unresolved problems within the pharmacy sector in Estonia. This could be connected to not having an actual national pharmaceutical policy [17]. One possible solution would be to learn from the experience of other EU countries as the questions and problems described in the monopoly or liberal pharmacy sector are similar in Estonia [2,5]. As some of the stakeholders in Estonia suggested a gradual transition period for pharmacy ownership and vertical integration restrictions, we could take a look at the Hungarian example. Similar restrictions on ownership were established there in 2011. By January 2014, institutional investors in existing pharmacies were obliged to appoint local pharmacists as directors of the pharmacy and sell at least 25% of their shares to the director or other private pharmacists. By 2017, investors will be obliged to sell at least 51% of their pharmacy shares to pharmacists. Similar to the ownership restrictions in Estonia, the maximum number of pharmacies that may be owned by one individual pharmacist is four, with the evident intention of outlawing pharmacy chains [18].

This study highlighted several other unsolved problems in the community pharmacy sector of Estonia. For example, changed ownership will not resolve the uneven geographical distribution of community pharmacies or the future role of pharmacies in the healthcare system. Lately, encouraging initiatives have been launched in developing professional standards, including the specification of professional roles for pharmacists and assistant pharmacists, and the development and harmonization of the community pharmacy services [19]. It seems that the representatives of pharmacy chains and professional pharmacy organizations have one common goal—the development of a contemporary and sustainable community pharmacy practice in Estonia.

While the sector is being reformed it would be wise first to identify the common ideas of all stakeholders, find ways for collaboration and not confront the main players of the community pharmacy sector. Otherwise, it could easily happen that main stakeholders dealing with a jumble of questions related to the new regulations forget the main purpose of healthcare for pharmacists, which is to provide quality pharmaceutical care to patients.

### Study Limitations

Important stakeholders in primary healthcare as governmental institutions, representatives of general practitioners and patients as well as representatives of the academia did not participate in the study and the results could therefore be biased. As such, the results may not be generalizable to the entire community pharmacy sector in Estonia. Many of the institutions and organizations explained their non-participation in the study with not having an official position regarding the restrictions of pharmacy ownership and vertical integration, or their lack of need for a study on this topic.

For data analysis, the text condensation method was used. This method helped to identify core information from open-ended replies. However, the condensation of meaning units was only possible using the positive/negative/neutral perceptions of the respondents as some of the questions received contrasting replies. However, this type of grouping could underline the impression of the stakeholders of the Estonian community pharmacy sector more as opponents than collaborators.

## 6. Conclusions

Pharmacy ownership and vertical integration restrictions have raised many questions and unclear expectations among different stakeholders in the pharmacy sector of Estonia. The study revealed two opposing positions regarding the future development of community pharmacies. Future owners underlined the need for increased professional independence and more healthcare-oriented operation of community pharmacies. Opponents of the reform argued against these ideas as community pharmacists do not have sufficient practical experience and finances to ensure the sustainable development of the community pharmacy sector. There is an urgent need for official government standpoints regarding the implementation of new regulations to assure the continuous provision of community pharmacy services and patient care in Estonia.

## Figures and Tables

**Table 1:** Pharmaceutical policy reforms in Estonia 1991–2015 ^a^.

Period	Reform Description
1991–1998	Privatization of community pharmacies; ownership of community pharmacies not restricted to pharmacists. Emergence of the first pharmacy chains connected to wholesale companies.
1996	Enactment of Medicinal Products Act, requirement to inform patients about the safe and appropriate use of medicines.
2002–2003	Development of main principles of pharmaceutical policy. Organization of Department of Medicines at the Ministry of Social Affairs. Introduction of generic prescribing.
2004	Estonia joined the European Union (EU).
2005–2006	Revision of the Medicinal Products Act to include a more detailed description of community pharmacy services. Introduction of geographic-demographic restrictions on the opening of new pharmacies.
2009	Introduction of digital prescriptions. Introduction of EU prescriptions.
2013	Opening of the first internet pharmacy. Repeal of establishment criteria for community pharmacies.
2014	Vertical integration restrictions, with a transition period of five years. Pharmacists became healthcare professionals.
2015	Measures undertaken to improve accessibility to medicines in rural areas: mobile pharmacy, grants for recently graduated specialists, requirement for pharmacy chains to open community pharmacies in rural areas if needed. Ownership restrictions for community pharmacies limited by the pharmacy profession, with a transition period of five years. Horizontal integration restrictions of up to four pharmacies, with a transition period of five years.

^a^: adapted from Volmer *et al.* [9].

**Table 2:** Positive and negative impact of the new regulations on the community pharmacy sector in Estonia.

Restrictions to Ownership of Community Pharmacies and Vertical Integration	
** Positive Impact**	**Negative Impact**
Pharmacist will be independent and able to make professional decisions not related to commercial interests.	Concerns about the sustainable development of community pharmacies as vertical integration helps to support non-profitable operation of retail sale of medicines.
A smaller number of pharmacies in towns means existing pharmacies will grow bigger, have more qualified staff, a larger selection of medicines and services.	Closing community pharmacies in rural regions could be seen as an imminent factor reducing accessibility to medicines.
Decrease in the prices of medicines due to the elimination of vertical integration, and the market opening to new wholesale companies.	Increase in medicine prices due to the restrictions on vertical integration that currently offers several discounts.
Community pharmacies can focus on the development of quality and patient-centered services.	Limited finances to educate pharmacists and invest in the development of community pharmacies. Community pharmacies might concentrate only on medicines and less on extended services.

## References

[1] Lluch M. (2009). Are regulations of community pharmacies in Europe questioning our pro-competitive policies?. Eurohealth.

[2] Vogler S. Liberalization in the pharmacy sector. *Competition Issues in the Distribution of Pharmaceuticals*, Proceedings of Session III of the Global Forum on Competition.

[3] Volkerink B., de Bas P., van Gorp N., Philipsen N. Study of regulatory restrictions in the field of pharmacies. http://ec.europa.eu/internal_market/services/docs/pharmacy/report_en.pdf.

[4] OECD Competition Issues in the Distribution of Pharmaceuticals (Contribution from Finland). Proceedings of Session III of the Global Forum on Competition.

[5] Lluch M., Kanavos P. (2010). Impact of regulation of community pharmacies on efficiency, access and equity. Evidence from the UK and Spain. Health Policy.

[6] Lai T., Habicht T., Kahur K., Reinap M., Kiivet R., van Ginneken E. (2013). Estonia: Health system review. Health Syst. Transit..

[7] Medicinal Products Act. https://www.riigiteataja.ee/en/eli/ee/Riigikogu/act/503092015003/consolide.

[8] Health Insurance Act. https://www.riigiteataja.ee/en/eli/ee/Riigikogu/act/503082015009/consolide.

[9] Volmer D., Bell J.S., Janno R., Raal A., Hamilton D.D., Airaksinen M.S. (2009). Change in public satisfaction with community pharmacy services in Tartu, Estonia, between 1993 and 2005. Res. Soc. Adm. Pharm..

[10] OECD Competition issues in the distribution of pharmaceuticals (Contribution from Estonia). Proceedings of Session III of the Global Forum on Competition.

[11] State Agency of Medicines Overview of the Activities of Estonian Pharmacies. 2015. http://www.sam.ee/sites/default/files/Review%20of%20activities%20of%20Estonian%20pharmacies_2014.pdf.

[12] Health Services Organization Act. https://www.riigiteataja.ee/en/eli/ee/Riigikogu/act/505032015002/consolide.

[13] Malterud K. (2012). Systematic text condensation: A strategy for qualitative analysis. Scand. J. Public Health.

[14] European Union Competition issues in the distribution of pharmaceuticals. Proceedings of Session III of the Global Forum on Competition.

[15] Wisell K., Winblad U., Sporrong S.K. (2015). Reregulation of the Swedish pharmacy sector—A qualitative content analysis of the political rationale. Health Policy.

[16] Volmer D., Vendla K., Vetka A., Bell J.S., Hamilton D. (2008). Pharmaceutical Care in Community Pharmacies: Practice and Research in Estonia. Ann. Pharmacother..

[17] Estonia Pharmaceutical Country Profile (2011). Published by the Ministry of Social Affairs in Collaboration with the World Health Organization. http://www.who.int/medicines/areas/coordination/estonia_pharmaceutical_profile.pdf.

[18] Eversheds (2015). Restrictions on Pharmacy Ownership in Hungary and the EU’s Recent Challenge against Them. http://www.eversheds.com/global/en/what/articles/index.page?ArticleID=en/Healthcare/Restrictions_on_pharmacy_ownership_in_Hungary_Feb2015.

[19] Keero A., Viidalepp A., Volmer D., Monvelt H., Kask H., Sarv K., Alamaa-Aas K., Sepp K., Saava L., Markov M. (2016). Quality Standards of Community Pharmacy Services.

